# Performance of a novel, urine-based test for the detection of cervical human papillomavirus infection

**DOI:** 10.1128/jcm.01318-25

**Published:** 2025-12-29

**Authors:** Sharmila Manjeshwar, Noah Kojima, Eric Tsang, David Pereira, Emmanuel Silva, Ha Duong, Felix Chao, Daniel R. Marshak, Ricky Y. T. Chiu, Jeffrey D. Klausner

**Affiliations:** 1 Phase Scientific Americas, Garden Grove, California, USA; 2 Department of Medicine, University of California Los Angeles 5116, Los Angeles, California, USA; 3 Phase Scientific International Ltd., Sha Tin, Hong Kong; 4 Keck School of Medicine, University of Southern California 5116, Los Angeles, California, USA; Mayo Clinic Minnesota, Rochester, Minnesota, USA

**Keywords:** high-risk HPV, self-collection, urine, PHASiFY technology, real-time PCR

## Abstract

Cervical cancer screening using the Pap smear is limited by invasive sampling and low participation among select populations. As human papillomavirus (HPV) is the leading cause of cervical cancer, urine-based testing offers a non-invasive alternative with the potential to increase overall screening participation. We developed and evaluated the performance of the Phase HPV Urine Test, a qualitative real-time PCR assay for the detection of 14 high-risk HPV (HR-HPV) subtypes in first-void urine samples. The assay utilizes a proprietary, room-temperature-stable sample collection system and a novel PHASiFY DNA extraction system optimized for large-volume (40 mL) urine processing. The analytical performance was evaluated to determine accuracy, precision, limit of detection (LOD), cross-reactivity, interference, and sample stability. Analytical performance was also assessed using 128 paired urine and clinician-collected cervical specimens (cervical broom in BD SurePath) tested with the Roche cobas 4800 HPV Test as the reference assay. The assay demonstrated 82.3% initial overall percent agreement with the cobas 4800 HPV test, improving to 93% after adjudication with HPV next-generation sequencing. The assay exhibited high precision (≥97.7%), a low LOD (15–30 viral copies/mL), interference with *C. albicans* and *T. vaginalis*, whole blood and peripheral blood mononuclear cells. Urine samples remained stable at room temperature for 10 days, with 95% concordance in results. The Phase HPV Urine Test provides accurate, reproducible, and non-invasive detection of HR-HPV from self-collected urine samples, supporting its potential for expanding access to cervical cancer screening.

**IMPORTANCE:**

Urine-based testing for high-risk human papillomavirus (HPV) provides a non-invasive, easily accessible alternative that may improve cervical cancer screening participation, especially among under-screened and underserved populations. The Phase HPV Urine Test combines a proprietary urine collection device with a novel large-volume DNA extraction system to enhance the sensitivity and reliability of HPV detection. Our study demonstrates that this approach yields high concordance with standard-of-care testing and maintains sample stability at ambient temperature, enabling flexible, cost-effective transport. By simplifying and standardizing urine collection and by expanding access beyond traditional clinical settings, this technology has the potential to increase uptake of HPV screening programs by overcoming barriers associated with invasive sampling methods and thereby reduce inequities. More importantly, these methods can contribute to earlier detection and prevention of cervical cancer.

## INTRODUCTION

Cervical cancer, largely caused by persistent infection with high-risk human papillomavirus (HR-HPV) subtypes, remains a significant public health problem in the United States and is the fourth most common cancer worldwide (1, 2), despite being largely preventable through vaccination and screening. In the United States, an estimated 13,800 new cases of invasive cervical cancer are diagnosed annually, with approximately 4,360 women dying annually (3).

In the United States, HPV vaccination programs in both girls and boys aged 9–14 years as well as cervical cancer screening programs in women aged 21–65 years have been implemented to prevent cervical cancer (4). The approved cervical cancer screening methods include a physician-collected endocervical specimen that is used for cytological evaluation (Pap smear) as well as for HPV testing by one of several FDA-approved, molecular HPV detection tests.

Although compliance with cervical cancer screening is relatively high at 73% of eligible women participating, there are considerable disparities in uptake among lower socioeconomic backgrounds, certain cultural minorities, and other underserved populations. The major barriers to screening adherence include (i) limited access to medical providers or lack of healthare insurance coverage (5) and (ii) various personal and system-level barriers to invasive cervical specimen collection (6). Therefore, a major focus of the U.S. Department of Health and Human Services (HHS) Healthy People 2030 program is to increase screening compliance (7). An important tool to increase screening compliance may be an HPV test that is widely accessible and offers ease of sample collection.

In this context, self-collected vaginal swab specimens collected in healthcare settings (or self-collected at home using Teal Wand) provide an alternative to a physician-collected, invasive endocervical specimens for HPV testing and were approved in the United States by the FDA in 2024. On the other hand, urine as an alternative sample type to cervical specimens for HPV detection has been investigated for over a decade. Importantly, the ease and convenience of urine sample collection may help alleviate social, ethnic, and personal aversion barriers to cervical specimen-based screening, resulting in higher compliance, improved coverage, and hence better patient outcomes (6, 8). Several studies have evaluated the feasibility and diagnostic accuracy of urine as a sample type with PCR-based methods for HPV detection, with varying results (9–13). Key limitations have been the lack of standardization in the urine sample collection method, the volume of urine collected, and the volume of urine used for HPV testing, which result in only moderate-to-good agreement with reference methods. Indeed, some recent studies show increased agreement with cervical specimen-based HPV detection when using a standardized method for the collection of first void urine (14–19).

We developed and evaluated the performance of a novel, qualitative molecular *in vitro* test (Phase HPV Urine Test) for the detection and genotyping of 14 high-risk HPV (HR-HPV) subtypes in first void urine specimens (defined as initial urine stream passed after a period without urinating, collected at any time of day), self-collected at home or at a physician’s office. Specimens were sent to the Phase Scientific Medical Laboratory (CLIA/CAP-accredited laboratory, Irvine, CA, USA) for processing and HPV testing.

## MATERIALS AND METHODS

### Phase HPV Urine Test

The Phase HPV Urine Test is a qualitative, real-time PCR assay designed to detect 14 HR-HPV subtypes (HPV16, HPV18, HPV31, HPV33, HPV35, HPV39, HPV45, HPV51, HPV52, HPV56, HPV58, HPV59, HPV66, and HPV68) with concurrent, individual typing of HPV16 and HPV18 DNA and grouped results for the remaining 12 HR-HPV subtypes in first-void urine. The test is performed using three proprietary kits for (i) urine sample collection, (ii) urine DNA extraction, and (iii) HPV PCR using the urine DNA by Real Time PCR on the QuantStudio 5 Dx platform (ThermoFisher Scientific, Waltham, MA, USA). All kits were developed under design control procedures and manufactured under standard GMP procedures in an ISO 13485 certified facility at Phase Scientific International Limited (Shenzhen, China). Three different lots of each of the kits were used to demonstrate the reproducibility and reliability of the test.

The unique features of the test include (i) proprietary urine collection kit that allows self-collection of up to 100 mL urine with 10-day stability at ambient temperature, enabling easy transportation at lower cost across diverse geographical regions, and (ii) novel DNA extraction method using proprietary PHASiFY technology that is able to concentrate and purify DNA from large volumes of biofluid (i.e., 40 mL urine), thus enabling detection of low levels of analytes/biomarkers.

### Urine collection kit

The sample collection kit consists of a labeled 120 mL urine transport bottle containing 5 mL of a proprietary preservative and a urine collection cup. The components are packaged in a biohazard bag, and the bag is packaged in a reusable shipping box. Internal design verification studies demonstrated that the preservative stabilizes HPV DNA for up to 10 days at room temperature, allowing for easy shipment of specimens. The kits were shipped to the four study sites (for clinical sample collections) where the patient collected up to 100 mL of urine using the cup, added the urine into the bottle, and mixed it with the preservative. Collected specimens were shipped back to the Phase Scientific Medical Laboratory (CLIA/CAP-accredited laboratory) at ambient temperature using the pre-authorized shipping label included in the kit. Samples once received in the laboratory were stored at room temperature until they were processed for DNA extraction.

### Urine DNA extraction kit

The Urine DNA extraction kit uses the proprietary aqueous two-phase system (ATPS) technology (PHASiFY) to extract and concentrate nucleic acid from 40 mL urine. The remaining urine sample was retained as a backup if necessary (stored at room temperature and used for repeat extraction or a second extraction at day 10 in the stability study). After concentrating the DNA through two ATPS buffer systems, the DNA was further purified and concentrated using a magnetic bead-based purification process performed in deep-well plates on the Tianlong GeneRotex 48 instrument (an automatic nucleic acid extractor from Xi’an Tianlong Science and Technology Co., Ltd., Shaanxi, China). The DNA was eluted in 60 µL DNAse/RNAse-free, PCR-grade water (Ambion/ThermoFisher Scientific, Carlsbad, CA, USA).

### HR-HPV PCR test kit

The HR-HPV PCR Kit comprises a PCR Master mix, a primer-probe mix (with forward and reverse primers to the L1 region of HPV genome as well as fluorescent probes without quenchers) for all 14 HR-HPV genotypes listed above as well as an internal control (β-globin) to ensure sample adequacy and optimal amplification. The kit also includes two external controls, one positive and one negative, that are amplified along with patient specimens in the same run. The real-time PCR run (45 cycles) is performed on the QuantStudio 5 Dx instrument (ThermoFisher Scientific) with reporter dyes assigned to the HPV subtypes as follows: Cy5 for HPV16, ROX for HPV18, FAM for HPV12+ (12 subtypes not differentiated), and VIC for internal control.

### PCR data analysis

After the run is complete, the real-time PCR run is analyzed by applying validated baseline and threshold settings, and the raw data are exported into a locked validated worksheet that analyzes run control status and contains analytical cycle threshold (Ct) cut-offs to evaluate sample validity (internal control β-globin, IC) for each of the samples and assign qualitative positive/negative status for each of the HPV subtypes (HPV16, HPV18, and HPV12+) in valid samples. The test output thus provides a qualitative (positive/negative) differentiated result for the HPV16 and HPV18 subtypes and grouped results for the remaining 12 HR-HPV subtypes (HPV12+).

### Clinical study and sample collection

An IRB-approved, prospective, clinical study in an enriched population was conducted at four different enrollment sites (Santa Fe, NM; Albuquerque, NM; El Paso, TX; and Salt Lake City, UT). The enriched population refers to a population in sites chosen because of the presence of a higher number of unvaccinated participants and higher prevalence of HPV in the area, but where participants were not pre-screened for HPV positivity. A total of 128 women, 18 years and older, were enrolled after providing informed consent. Demographic information was collected from all participants; however, women who were menstruating at the time of enrollment were excluded from the study. All enrolled participants provided a physician-collected cervical specimen (cervical broom in BD SurePath, BD) and a patient-self-collected, first-void urine specimen. For the urine collection, enrolled participants were asked to self-collect the urine sample only if they had not passed urine for 2 h prior, or if they had, then to wait at least one more hour before providing the urine sample. The first-void urine specimen refers to 60–100 mL of urine (always including the initial, first-stream) collected at any time of day during their visit to the collection site if the previous criterion was met. Urine was first collected in the collection cup and then poured into the urine transport bottle containing the preservative. The urine specimens were shipped at ambient temperature to the Phase Scientific Medical Laboratory for specimen processing and testing with the HPV urine test. A first DNA extraction from 40 mL urine using the PHASiFY DNA extraction kit was performed the day the sample was received in the laboratory, and a second extraction (based on remaining urine volume) was performed at day 10 (from date of collection) for sample stability studies (samples were stored at room temperature until extraction). The endocervical specimen was subjected to standard of care HPV testing by the FDA-approved, Roche cobas 4800 HPV PCR Test in an independent CLIA-certified laboratory, and the results were used as the reference test.

### Contrived specimens

Individual donor urine from HPV-uninfected women was purchased from Medix Biochemica (Westbrook, ME, USA) for use in preparing spike-in specimens. Positive contrived specimens (*n* = 55) were prepared by spiking the 14 HR-HPV subtype plasmids (ATCC, Maryland, USA or Genewell HPV gDNA Reference Standard set, Genewell, Shenzhen, China) individually, at three to four different concentrations from low, that is, near limit of detection (LOD) to high (at 10^6^, 10^5^, 10^4^, and 10^3^ copies in 40 mL urine). In addition, “mixed” specimens were prepared by spiking in a mix of HPV plasmids (combinations of HPV16, HPV18, and one of the HPV12+ plasmids) at a low level. The plasmids from ATCC and the Genewell reference standard set are quantified with droplet digital PCR (average dPCR copies/mL) by the manufacturer, and Certificates of Analysis for each plasmid and each lot were obtained and used to determine the number of copies required to spike-in. Contrived negative specimens included the individual, HPV negative, female donor urine specimens (*n* = 9) used for all the spike-in studies.

### Analytical accuracy

A total of 191 specimens were tested in this study, which included 63 contrived specimens and 128 clinical specimens. All samples were tested in the HPV urine test. For the clinical specimens, the first DNA extraction, performed the day they were received in the laboratory, was used in the accuracy study. For the clinical samples, the phase HPV PCR assay result was compared with the cobas HPV test as the assay reference. For both contrived and clinical samples, the overall percent agreement (OPA%) with expected results (contrived specimens) and cobas HPV test (clinical specimens) as well as confidence intervals was calculated using MS Excel functions.

### Precision

For the precision study, a subset of 30 specimens from the accuracy study was chosen. These included: (i) 20 contrived specimens spiked-in with individual HPV plasmids at high and low levels or a mix of HPV plasmids and (ii) 10 clinical samples both positive and negative for HPV (based on cobas HPV reference test) were tested. The 30 specimens also represented specimens collected in three different lots of Urine Collection kits, extracted with three different lots of PHASiFY DNA Extraction kits, tested with three different HR-HPV PCR kits, and all testing was performed on two different QuantStudio 5 Dx instruments by four operators over 5 days (inter-assay precision) to demonstrate reproducibility and reliability of the assay. For intra-assay precision, the 30 specimens were tested in triplicate in the same run.

### HPV subtype determination by next-generation sequencing (NGS)

Samples from the accuracy study that were not concordant with the cobas reference test result were adjudicated with a third independent test, HPV NGS, available at CD Genomics (Shirley, NY, USA). Due to the lack of FDA-approved sequencing methods, an in-house developed/validated custom HPV NGS panel by CD Genomics was chosen as the best alternative. CD Genomics is an ISO 9001 certified facility that provides HPV NGS sequencing service with HPV subtype determination. An aliquot of the same DNA that was used in the HR-HPV PCR assay was sent for testing with the NGS assay. The NGS assay uses 500 ng DNA for library preparation combined with a paired-end (PE 150), hybrid capture-based sequencing method that is run on the Illumina NovaSeq platform with a mean sequencing depth of >200× and a sensitivity of 0.5%. The raw NGS data were processed using proprietary algorithms to assign the HPV subtype.

### LOD study

For each plasmid (HPV16, HPV18, and HPV31), a series of six dilutions with at least one concentration level above LOD and one level below LOD (preliminary LOD determined in prior study, data not shown), for each plasmid was spiked into HPV-negative urine. One negative (no HPV plasmid spike-in) urine specimen was also included. Three separate extractions (Runs 1, 2, and 3 using three different lots of PHASiFY DNA Extraction kits) were performed for each dilution series of each plasmid. The extracted DNA for each dilution series for each plasmid was tested in 12 replicates using one lot of HR-HPV PCR kit. The PCR data for all 12 replicates for each concentration in the series in each lot for each plasmid were subjected to Probit Analysis and regression analysis using MS Excel and were used to compute the C95 concentration for each extraction of the three HPV plasmids as well as to set the LOD.

### Cross-reactivity/specificity study

Six microorganisms, two HPV related (HPV6 and HPV11, included in the Genewell Reference standard set), and four unrelated organisms from ATCC (Manassas, VA, USA) (*Chlamydia trachomatis*, *Neisseria gonorrhea, Candida albicans* and *Trichomonas vaginalis*) that can be present in female urine were spiked (at one level, 1 × 10^6^ copies/40 mL urine) into negative female donor urine in the absence or presence of HPV16, HPV18 or HPV31 (at LOD or 2× LOD) plasmids and subjected to the Phase HPV Urine Test.

### Interfering substances study

The study was designed to determine whether exogenous substances (feminine hygiene products, vaginal lubricant, and douche), medications (clotrimazole), and endogenous substances (whole blood [Innovative Research, Novi, MI], peripheral blood mononuclear cells [ATCC], and glucose [Sigma-Aldrich, St. Louis, MO, USA]) that may be present concomitantly with HPV in female urine interfered with the detection of HPV. Six substances were spiked at one concentration level (≥1% vol/vol or ≥1% wt/vol) into HPV negative urine in the absence or presence of HPV16, HPV18, or HPV31 plasmids (at LOD or 2× LOD) and subjected to the Phase HPV Urine Test.

### Urine specimen stability

To validate the stability of the urine collection kit in a clinical testing workflow, a total of 40 patient specimens (20 HR-HPV-positive and 20 HPV-negative specimens) from the accuracy study were extracted first upon receipt in the laboratory (DNA1). The remaining specimen was stored at room temperature until day 10 (from the recorded collection date), and a second DNA extraction (DNA2) was performed. Both the initial and day 10 extracted DNA samples for each patient were tested with the Phase HPV Urine Test. The HPV status (positive/negative) and HPV subtype identity were compared between DNA1 and DNA2 for each specimen and compared with cobas HPV result.

## RESULTS

### Clinical study population

A total of 128 women between the ages of 19 and 77 years were enrolled from May to August 2024 at four sites (Santa Fe, NM; Albuquerque, NM; El Paso, TX; and Salt Lake City, UT). The median age of participants was 36 years and 28.1% of the population was 18–29 years of age. Greater than 65% of the participants were White/Hispanic or Latino, and 22.7% were White/non-Hispanic or Latino. Detailed demographics are presented in Table S1.

### Analytical accuracy and precision

#### Contrived specimens

All contrived specimens were processed and tested with the Phase HPV Urine Test. Of these, 61 out of 63 specimens (96.8%) were concordant with expected results (Table S2). Two specimens failed to amplify one of the two spiked HPV plasmids. Of these, one sample was spiked with one of the HPV12+ and HPV16 plasmids at 1,200 cp of each plasmid in 40 mL urine and another was spiked with one of the HPV12+ and HPV18 plasmids at 1,200 cp of each plasmid in 40 mL urine. In each case, the HPV12+ was detected, but the HPV16 or HPV18 was not detected.

#### Clinical specimens

Urine specimens from all participants (*n* = 128) were processed upon arrival in the laboratory and subjected to the Phase HPV Urine Test. The results were compared with the Roche cobas HPV Test result (as the reference test, obtained with the paired endocervical specimen) and are summarized in Table 1. The distribution of HPV positive samples (by the reference cobas HPV test) in the study includes 3 HPV16-positive, 2 HPV18-positive, and 30 HPV12+ positive samples with no co-detections observed by the reference cobas HPV test (refer to Table S1). Given the small numbers of HPV16- and HPV18-positive samples, analysis by subtype was not performed, and the data is represented as OPA (analyzed with HPV16, HPV18, and HPV12+ combined). Therefore, the initial OPA was 82.3%. As can be seen from Table 1, 22 of the 128 clinical samples were not concordant, with 15 samples urine positive/cobas negative (potentially false positive) and 7 samples urine negative/cobas positive (potentially false negative), based on the result of the reference cobas HPV test. Therefore, non-concordant samples were subjected to a third independent test, HPV NGS (CD Genomics) for adjudication. Of the specimens, 14 urine-positive/cobas-negative and 4 urine-negative/cobas-positive samples were subjected to HPV NGS testing; 4 samples were not sequenced due to insufficient DNA for NGS testing (refer to Table 2). Based on the sequencing data presented in Table 2, 13 samples were re-assigned as concordant with the Phase HPV Urine Test (with an additional 11 true positives and 2 true negatives), thereby showing improved assay performance with an adjusted % OPA of 93.0% (Table 1) with 5 samples (S003, S059, S081, S119, and [S098-not sequenced] remaining false positive and 4 samples (S106 and [S037, S043, and S063 not sequenced]) remaining false negative (Table 2).

**TABLE 1:** Agreement between Phase HPV Urine Test and Roche cobas HPV results

	Roche cobas HPV (cervical specimen)^*b*^		%Agreement (95% CI^*c*^: lower, upper)			
Phase HPV Urine Test^*a*^	Positive	Negative	Overall	Positive	Negative	Cohen’s kappa
Initial OPA^*d*^ (%)						
Positive	26	16	82.3 (76.3, 89.4)	81.3	83.3	0.58
Negative	6	80				
Adjusted OPA^*e*^ (%)						
Positive	37	5	93.0 (88.5, 97.4)	90.2	94.3	0.84
Negative	4	82				

^*a*^ HPV urine test performed using 40 mL urine sample obtained from participants (*n* = 128) enrolled in the clinical cohort as part of the Accuracy study.

^*b*^ Cobas HPV Test performed at an independent clinical laboratory on physician-collected cervical specimen collected from the patient at the same time as the urine specimen.

^*c*^ CI, confidence interval.

^*d*^ OPA, overall percent agreement.

^*e*^ Adjusted OPA, adjusted overall percent agreement obtained by re-assigning HPV positive/negative samples after adjudicating HPV next-generation sequencing (NGS) result with HPV urine test result shown in Table 2.

**TABLE 2:** Comparison of Phase HPV Urine Test with sequencing results

Sample ID	Phase HPV Urine Test^*a*^ result	Cobas HPV^*b*^ result	Sequencing^*c*^ result
Urine positive/cobas negative samples			
S101	HPV12+, HPV16	HPV12+	HPV56 (HPV12+)/HPV16
S103	HPV12+	Negative	HPV68 (HPV12+)
S003	HPV12+	Negative	Negative
S013	HPV12+	Negative	HPV51 (HPV12+)
S024	HPV12+	Negative	HPV68 (HPV12+)
S050	HPV12+	Negative	HPV51 (HPV12+)
S059	HPV12+	Negative	Negative
S062	HPV12+	Negative	HPV68 (HPV12+)
S073	HPV12+	Negative	HPV68 (HPV12+)
S079	HPV 16	Negative	HPV 16
S081	HPV12+, HPV16	Negative	HPV 16
S094	HPV12+	Negative	HPV56 (HPV12+)
S115	HPV12+	Negative	HPV68 (HPV12+)
S119	HPV12+	Negative	Negative
S098	HPV12+, HPV18	HPV12+	NS^*d*^
Urine negative/cobas positive samples			
S106	Negative	HPV12+	HPV45 (HPV12+)
S065	Negative	HPV12+	Negative
S096	Neg 12+, HPV 16	HPV12+	HPV 16
S129	Negative	HPV 16	Negative
S037	Negative	HPV12+	NS^*d*^
S043	Negative	HPV12+	NS^*d*^
S063	Negative	HPV12+	NS^*d*^

^*a*^ HPV urine test performed using 40 mL urine sample obtained from patient.

^*b*^ Cobas HPV test performed at an independent clinical laboratory on physician-collected cervical specimen collected from the patient at the same time as the urine specimen.

^*c*^ HPV next-generation sequencing test performed at an independent laboratory on an aliquot of the urine DNA tested with the Phase HPV Urine Test.

^*d*^ NS refers to samples that were not sequenced due to insufficient DNA.

### Precision

Precision was assessed with 20 contrived specimens and 10 clinical samples either positive or negative for HPV. Testing incorporated three different lots of urine collection kits, DNA extraction kits, and PCR kits, and was performed on two QuantStudio 5 Dx instruments by four operators over 5 days to demonstrate reproducibility and robustness of the assay. The results summarized in Table 3 show that the Phase HPV Urine Test is reproducible and reliable with a precision of >95%. The inter-assay precision was 98.6% (with the positive agreement being 97.9%) and the intra-assay precision was 97.7% (with the positive agreement being 96.5%). The negative agreement was 100% in both precision studies.

**TABLE 3:** Precision and reproducibility^*a*^ of the Phase HPV Urine Test

Inter-assay precision			Intra-assay precision		
	Expected HPV Result^*b*^			Expected HPV result^*b*^	
Phase HPV Urine Test	HPV positive	HPV negative	Phase HPV Urine Test	HPV positive	HPV negative
Positive	93	0	Positive	55	0
Negative	2	50	Negative	2	30
% Precision	98.6%		% Precision	97.7%	
% Positive agreement	97.9%		% Positive agreement	96.5%	
% Negative agreement	100%		% Negative agreement	100%	

^*a*^ The precision study, including reproducibility, was performed as described in Materials and Methods on a subset of specimens (*n* = 30) from the Accuracy Study (Table 1; Table S2) including both contrived (spiked in) and clinical samples (HPV-positive and HPV-negative). Qualitative HPV result for each sample was compared between runs (inter-assay) and in-between run (Intra-assay) as well as to the expected result and summarized in the table.

^*b*^ Expected result is either the cobas HPV test result (for clinical specimens) or the HPV plasmid(s) spiked in.

### LOD

The LOD results for HPV16, HPV18, and HPV31 (representative of HPV12+) showed that the concentration where 100% detection is observed for each plasmid was: HPV16 = 30 copies/mL (1,200 copies/40 mL) urine; HPV18 = 15 copies/mL (600 copies/40 mL) urine; and HPV12+ (HPV31 as a representative) = 15 copies/mL (600 copies/40 mL) urine (Table S3).

### Analytical specificity—cross-reactivity and interfering substances

The qualitative HPV PCR results obtained from cross-reactivity and interfering substances studies are presented in Table 4. Most cross-reactants and interfering substances did not affect HPV detection even at a low level (i.e., at LOD [HPV16] or at 2× LOD [HPV18 HPV31]) and showed no effect on the detection of the internal control. Notable exceptions include the interference observed with *C. albicans* and *T. vaginalis,* both of which inhibited the detection of all three HPV subtypes. Of the interfering substances tested, clotrimazole, glucose, or feminine products (vaginal lubricant or douche) did not interfere with HPV detection. However, as expected, whole blood did interfere either partially (at 1% vol/vol) or completely (at 4% vol/vol) with HPV subtype detection, as did PBMC at a concentration of 1 × 10^6^ cells.

**TABLE 4:** Phase HPV Urine Test—Cross-reactivity and interfering substances affecting HPV detection

Cross-reactant^*a*^ or interfering substance^*a*^ tested	Detection of internal control	HPV subtype			Cross-reactivity or interference observed
		HPV12+ (HPV31)	HPV16	HPV18	
HPV6 (1 × 10^6^ copies)	Detected	Detected	Detected	Detected	None
HPV11 (1 × 10^6^ copies)	Detected	Detected	Detected	Detected	None
*C. trachomatis* (1 × 10^6^ copies)	Detected	Detected	Detected	Detected	None
*N. gonorrhoeae* (1 × 10^6^ copies)	Detected	Detected	Detected	Detected	None
*C. albicans* (1 × 10^6^ copies)	Not detected	Not detected	Not detected	Not detected	Yes, complete
*T. vaginalis* (1 × 10^6^ copies)	Detected	Not detected	Not detected	Not detected	Yes, complete
Whole blood 4%	Detected	Not detected	Not detected	Not detected	Yes, complete
Whole blood 1%	Detected	Detected	Not detected	Detected	Partial interference
PBMC^*b*^ (1 × 10^6^ cells)	Detected	Not detected	Not detected	Not detected	Yes, complete
Glucose 1% (wt/vol)	Detected	Detected	Detected	Detected	None
Clotrimazole 1% (wt/vol)	Detected	Detected	Detected	Detected	None
Vaginal lubricant (1%) (vol/vol)	Detected	Detected	Detected	Detected	None
Feminine douche 1% (wt/vol)	Detected	Detected	Detected	Detected	None

^*a*^ Six cross reacting organisms (HPV related and unrelated) and six interfering substances were spiked individually into 40 mL urine at the levels indicated in the table. In separate 40 mL aliquots, the same organisms or substances (at the levels indicated in the table) were co-spiked along with one of three HPV plasmids (HPV16, HPV18, or HPV31) at 1,200 copies (at LOD or 2× LOD). All samples were subjected to HPV urine test and the qualitative (HPV subtype detected/not detected) is presented in the table.

^*b*^ PBMC, peripheral blood mononuclear cells.

### Urine sample stability for HPV detection

The comparison between the initial and day 10 extracted DNA samples from 40 urine specimens (negative or positive for HPV) showed that 95% (38/40) of samples were concordant between the initial and day 10 as well as with expected cobas HPV result (Table S4). Of the HPV-positive samples, 18 out of 20 were positive at day 10 with a non-significant shift in Ct values for the HPV subtype (data not shown). Of the 18 samples, 16 samples showed a Ct change of less than 1 and the remaining 2 samples showed a change greater than 1 Ct but less than 1.5 Ct (or <0.5 log). In addition, all 40 specimens (20 positive and 20 negative) showed 100% detection of the internal control (data not shown), which confirms the robust performance of the urine collection kit (to preserve DNA) and supports HPV detection even at 10 days post collection.

## DISCUSSION

This study presents the first performance evaluation and validation of the Phase HPV Urine Test, a novel, non-invasive molecular diagnostic designed to detect HR-HPV subtypes from first-void urine, in a clinical laboratory following guidelines for qualitative high complexity tests. Our findings demonstrate that the combination of a proprietary ambient-stable urine collection kit and PHASiFY large-volume DNA extraction applied to a downstream PCR assay offers a viable alternative to traditional, gold-standard, cervical specimen-based testing.

A key strength of the Phase HPV Urine Test is that all its components, from sample collection to the PCR assay, were internally developed and optimized for the urine sample type. Previous studies that evaluate urine-based HPV detection use existing FDA-approved methodologies which restrict modifications to the PCR method parameters (i.e., the assay Ct cut-off), despite the clear need to optimize such parameters for the urine sample type (15, 16). Our studies, although promising (initial agreement = 82.3%, with sequencing adjudication = 93.2%), also indicate the need to refine the PCR cycle threshold cut-offs, especially for the HPV12+ channel in larger clinical studies with histology confirmed end point data to help improve the accuracy of the test. Importantly, this study highlights potential sensitivity advantages of the Phase HPV Urine Test over other methods for HPV DNA testing in self-collected urine specimens, an area historically limited by sample dilution and inefficient DNA recovery.

Several other factors also potentially contribute to the performance of the Phase HPV Urine Test. By performing NGS, we were able to confirm that many of the originally apparent HPV12+ false positives were not due to lack of specificity of the phase HR-HPV PCR assay, but rather due to potentially more sensitive detection of HPV compared to the reference test. This further emphasizes the need for further large-scale validation to establish our own clinical cut-offs. A second contributing factor is the low-cost, ambient-stable urine collection kit, which can efficiently preserve low levels of analyte at room temperature.

Another contributing factor is the PHASiFY technology, which allows extraction and concentration of DNA from large volumes and alleviates the technical concern that urine samples >20 mL may be too diluted and reduce sensitivity for HPV detection (11). The proprietary PHASiFY DNA purification technology employs an ATPS to purify and concentrate DNA of various sizes from large volumes of biological samples (i.e., urine and plasma). The PHASiFY technology is coupled with DNA precipitation or magnetic bead-based DNA purification to further purify and concentrate DNA. The performance of PHASiFY technology was initially demonstrated in a study which showed that it outperformed conventional column-based purification methods in extracting circulating, cell-free DNA (cfDNA) from plasma obtained from cancer patients, allowing for the detection of low-level mutations that were not previously detectable by droplet digital PCR (20). With PHASiFY, the DNA extracted from a single urine sample can be leveraged for multiple assays (i.e., HPV testing combined with molecular testing for sexually transmitted infections or women’s health), particularly for biological targets that rely on a cervical/vaginal specimen due to the lower sensitivity in urine using current urine collection and nucleic acid extraction methods. In addition, the higher yields of DNA are suitable and sufficient for discovery applications or those that require large DNA quantities such as methylation studies or NGS. This efficiency will allow the use of the extracted DNA for multiple tests in the clinical laboratory, improving sample utility and reducing operational costs. Indeed, most of the clinical samples that were sequenced for adjudication had sufficient remaining DNA for NGS library preparation.

Another key strength of the Phase HPV Urine Test is its logistical robustness. The proprietary urine collection device maintains DNA stability for at least 10 days at ambient temperature, reducing the burden of cold-chain logistics and increasing suitability for home-based or remote screening applications. Furthermore, urine samples could be collected at home and sent by mail to the clinical laboratory for HPV testing, thereby removing the need for an initial clinic-based pelvic examination. This feature aligns with current U.S. public health initiatives, such as Healthy People 2030, aimed at increasing cervical screening participation by 2030. Non-invasive urine samples seem to be preferred by women over both provider-collected cervical samples and self-collected cervicovaginal samples for HPV testing (21–23). Precision studies confirmed assay reproducibility across multiple variables including operators, instruments, and reagent lots. The established analytical sensitivity (LOD 15–30 copies/mL) suggests that the assay is suitable for early-stage detection of HPV infections, a critical factor for effective screening. The assay also demonstrated minimal cross-reactivity or interference from common urinary contaminants and co-infections, with the exception of whole blood, *C. albicans,* and *T. vaginalis,* which warrant further investigation. The interference of whole blood suggests that urine collection for HPV detection should be avoided during menstruation or in the presence of other medical conditions that result in hematuria.

Notwithstanding these advantages, this study has some limitations. First, although the spike-in studies were performed with high quality viral DNA (i.e. quantified by digital PCR or qPCR), data with live virus or virus-like particles are not currently available and will be performed as part of future product development studies. Secondly, a limitation of the study is the interference seen with *C. albicans* and *T. vaginalis* at 1E+06 copies/40 mL. Although additional data are not available at the present time, future product development studies are planned to investigate and minimize the interference. Finally, the clinical sample set was relatively small and lacked sufficient HPV16 and HPV18 positive samples (*n* = 5, by cobas reference test), which restricted subtype-specific analysis. This study evaluated analytical performance and provides preliminary evidence of concordance with a reference assay since it did not include histological clinical end point status (i.e., cervical intraepithelial neoplasia [CIN] status) for the study participants. Therefore, for broader clinical implementation, future studies with larger cohorts with histology-confirmed disease status will be essential to determine positivity rates and refine the clinical PCR cycle threshold cut-off, particularly for the HPV12+ pool to fully validate the clinical performance and clinical utility of the Phase HPV Urine Test. Nevertheless, the analytical evaluation presented here is informative and will be used to guide the planning of larger studies with clinical endpoint data. Additionally, expansion of the study population to include under-screened and diverse demographics will further establish the assay’s real-world impact. In general, urine-based HR-HPV tests have not yet been approved by the FDA for clinical use, and further studies to evaluate and to improve the accuracy of urine-based HR-HPV testing for CIN2+ detection in a primary screening population, including among women living with HIV, are needed.

### Conclusion

The Phase HPV Urine Test demonstrates high analytical performance, reproducibility, and concordance with standard-of-care methods, along with the advantages of non-invasive, self-collected urine sampling and ambient temperature stability. Therefore, this test provides a compelling, patient-friendly alternative to conventional cervical HPV testing, and, with further evaluation in larger clinical studies with histology-confirmed endpoints, has the potential to expand access to cervical cancer screening, particularly among under-screened populations. The ease of use of the urine collection kit, logistical advantages, and ability to extract and concentrate DNA from large volumes of urine position the Phase HPV Urine Test as a valuable tool in expanding access to cervical cancer screening in the United States and globally.

## Data Availability

The data presented in this study are available from the corresponding author upon reasonable request.

## SUPPLEMENTAL MATERIAL

Supplemental material jcm.01318-25-s0001.docx Supplemental results and Tables S1 to S4.

ASM does not own the copyrights to Supplemental Material that may be linked to, or accessed through, an article. The authors have granted ASM a non-exclusive, world-wide license to publish the Supplemental Material files. Please contact the corresponding author directly for reuse.
