# Supplementary material for: Performance of a novel, urine-based test for the detection of cervical human papillomavirus infection
Source: J Clin Microbiol. 2025 Dec 29;64(2):e01318-25. doi: 10.1128/jcm.01318-25 (PMC12893011; doi:10.1128/jcm.01318-25)
Supplement: Supplemental material — Supplemental results and Tables S1 to S4. [file jcm.01318-25-s0001.docx]

**Performance of a novel, urine-based test for detection of cervical human papillomavirus infection**

**AUTHORS**

Sharmila Manjeshwar^1^, Noah Kojima^2^, Eric Tsang^1^, David Pereira^1^, Emmanuel Silva^1^, Ha Duong^1^, Felix Chao^1^, Daniel R. Marshak^1^, Ricky Y.T. Chiu^3^, Jeffrey D. Klausner^4^

**AUTHOR AFFILIATIONS**

^1^Phase Scientific Americas, Garden Grove, California, USA

^2^Department of Medicine, University of California Los Angeles, Los Angeles, California, USA

^3^Phase Scientific International Ltd., Sha Tin, NT, Hong Kong

^4^Keck School of Medicine, University of Southern California, Los Angeles, California, USA

**Corresponding Author:**

Sharmila Manjeshwar, PhD, MB^(ASCP)^, CGMBS

sharmila.manjeshwar@phasesci.com

**Supplemental Text**

**Results:**

**Analytical Accuracy: Agreement of HPV subtype detection**:

Our data indicate that, of the urine HPV positive specimens, 2/3 urine HPV 16 positive samples, 2/2 urine HPV18 and 22/30 urine HPV12+ positive were 100% concordant with their reference cobas HPV test. An additional 2 urine HPV12+ were double positive (i.e. one was HPV12+/HPV16 positive and one was HPV12+/HPV18 positive) when the cobas reference test was only positive for HPV12+.

**Supplemental Tables**

| **Table S1. Clinical Study Subject Demographics** | | |  |  |  | |
| --- | --- | --- | --- | --- | --- | --- |
|  | **# of subjects** | **% of Total** | **# Positive by cobas reference method** | | | |
| **Age (years)** |  |  | **HPV12+** | **HPV16** | | **HPV18** |
| 19-29 | 36 | 28.1 | 12 | 1 | | 0 |
| 30-65 | 89 | 69.5 | 17 | 1 | | 2 |
| >65 | 3 | 2.3 | 1 | 1 | | 0 |
| **Race/Ethnicity** |  |  |  |  | |  |
| White/Hispanic or Latino | 84 | 65.6 |  |  | |  |
| White/non-Hispanic or Latino | 29 | 22.7 |  |  | |  |
| African American | 1 | 0.8 |  |  | |  |
| Native American | 3 | 2.3 |  |  | |  |
| Asian | 2 | 1.6 |  |  | |  |
| Mixed (White-Native American) | 1 | 0.8 |  |  | |  |
| unknown (did not provide) | 8 | 6.3 |  |  | |  |

^a^Participants were asked to complete a questionnaire (after obtaining written informed consent) and provide demographic information that is summarized in the Table by category.

^b^Subjects could report multiple responses.

| **TABLE S2. Accuracy of Phase HPV Urine Test (Contrived Specimens)** | | | | | |
| --- | --- | --- | --- | --- | --- |
|  | **Expected Result^b^** | | **%Agreement (95% CI)** | | |
| **Phase HPV Urine^a^ Test** | **Positive** | **Negative** | **Overall** | **Positive** | **Negative** |
| **Positive** | 53 | 0 | 96.8 (92.5, 101.15) | 96.3 | 100 |
| **Negative** | 2 | 8 |  |  |  |

^a^HPV Urine Test performed using 40mL urine samples spiked with one more of the 14 high-risk HPV to prepare the contrived specimens (part of Accuracy Study) as outlined in Materials and Methods. Observed Phase HPV Urine Test Result was compared with Expected Result (i.e. plasmid spiked in).

^b^Expected Result is based on HPV plasmid spiked in individually or mix of plasmids spiked in.

^c^CI, Confidence Interval

| **TABLE S3. Limit of Detection for HPV subtypes in 40mL urine** | | | | |
| --- | --- | --- | --- | --- |
| **HPV subtype** | **C95 Concentration for HPV sub-type^a^ (copies/mL)** | | | **LOD^b^ for HPV subtype (copies/mL)** |
|  | **Run 1** | **Run 2** | **Run 3** |  |
| **HPV16** | 15.8 | 13.0 | 13.2 | 16.0 |
| **HPV18** | 13.3 | 13.9 | 13.1 | 15.0 |
| **HPV31  (HPV12+)** | 10.2 | 9.4 | 12.2 | 15.0 |

^a^ LOD study was performed as described in Materials and Methods. Briefly, each plasmid was spiked in a 6-dilution series into 40mL urine plus one urine sample with no spike-in (negative). Each dilution series was performed 3 separate times and extracted with 3 different lots of Phasify DNA extraction kits (Run 1, 2, 3) and tested with one lot of Phase HPV PCR kit. C95 was computed and LOD determined for each plasmid, HPV31 is shown in the Table as a representative of HPV 12+.

^b^LOD, Limit of Detection.

| **TABLE S4. Sample Stability in Phase Urine Collection Kit** | | | |
| --- | --- | --- | --- |
| **Sample #** | **Phase HPV Urine Test Result** | | **cobas HPV Test^b^ Result** |
|  | **DNA 1^a^ (Initial)** | **DNA 2^a^**  **(Day 10)** |  |
| 1 | HPV12+ | HPV12+ | HPV12+ |
| 2 | Neg | Neg | Neg |
| 3 | HPV12+ | HPV12+ | HPV12+ |
| 4 | Neg | Neg | Neg |
| 5 | Neg | Neg | Neg |
| 6 | Neg | Neg | Neg |
| 7 | Neg | Neg | Neg |
| 8 | Neg | Neg | Neg |
| 9 | HPV12+ | HPV12+ | HPV12+ |
| 10 | HPV12+ | HPV12+ | HPV12+ |
| 11 | Neg | Neg | Neg |
| 12 | HPV12+ | HPV12+ | HPV12+ |
| 13 | HPV12+ | HPV12+ | HPV12+ |
| 14 | **Neg** | **Neg** | **HPV12+** |
| 15 | HPV12+ | HPV12+ | HPV12+ |
| 16 | HPV12+ | HPV12+ | HPV12+ |
| 17 | Neg | Neg | Neg |
| 18 | Neg | Neg | Neg |
| 19 | **Neg** | **Neg** | **HPV12+** |
| 20 | Neg | Neg | Neg |
| 21 | HPV12+ | HPV12+ | HPV12+ |
| 22 | HPV12+ | HPV12+ | HPV12+ |
| 23 | Neg | Neg | Neg |
| 24 | Neg | Neg | Neg |
| 25 | Neg | Neg | Neg |
| 26 | Neg | Neg | Neg |
| 27 | Neg | Neg | Neg |
| 28 | HPV 18 | HPV 18 | HPV 18 |
| 29 | HPV12+ | HPV12+ | HPV12+ |
| 30 | Neg | Neg | Neg |
| 31 | Neg | Neg | Neg |
| 32 | HPV12+ | HPV12+ | HPV12+ |
| 33 | HPV12+ | HPV12+ | HPV12+ |
| 34 | HPV12+ | HPV12+ | HPV12+ |
| 35 | Neg | Neg | Neg |
| 36 | Neg | Neg | Neg |
| 37 | HPV 16 | HPV 16 | HPV 16 |
| 38 | Neg | Neg | Neg |
| 39 | HPV 18 | HPV 18 | HPV 18 |
| 40 | HPV 16 | HPV 16 | HPV 16 |

^a^Forty participants from the Accuracy Clinical study (Table 1) that provided ≥100mL urine were included in this study. For the initial test, DNA (DNA1) was extracted from 40mL urine the day the sample was received in the laboratory. The remainder was stored at room temperature for 10 days when an additional 40mL was used to perform a 2^nd^ DNA extraction (DNA2). All DNA samples were subjected to the Phase HPV PCR Test. Data summarized in the Table shows concordance between DNA1, DNA2 and the cobas HPV result for each specimen.

^c^cobas HPV Test performed at an independent clinical laboratory on physician-collected cervical specimen collected from the patient at the same time as the urine specimen.
